# Characterization of the Complete Mitochondrial Genome of *Harpalus sinicus* and Its Implications for Phylogenetic Analyses

**DOI:** 10.3390/genes10090724

**Published:** 2019-09-18

**Authors:** Xiaolei Yu, Wei Tan, Huanyu Zhang, Weiling Jiang, Han Gao, Wenxiu Wang, Yuxia Liu, Yu Wang, Xiaoxuan Tian

**Affiliations:** 1 Tianjin State Key Laboratory of Modern Chinese Medicine, Tianjin University of Traditional Chinese Medicine, Tianjin 301617, China; 2 School of Integrative Medicine, Tianjin University of Traditional Chinese Medicine, Tianjin 301617, China

**Keywords:** *Harpalus sinicus*, mitochondrial genome, phylogeny

## Abstract

In this study, we report the complete mitochondrial genome of *Harpalus sinicus* (occasionally named as the Chinese ground beetle) which is the first mitochondrial genome for *Harpalus*. The mitogenome is 16,521 bp in length, comprising 37 genes, and a control region. The A + T content of the mitogenome is as high as 80.6%. A mitochondrial origins of light-strand replication (OL)-like region is found firstly in the insect mitogenome, which can form a stem-loop hairpin structure. Thirteen protein-coding genes (PCGs) share high homology, and all of them are under purifying selection. All tRNA genes (tRNAs) can be folded into the classic cloverleaf secondary structures except *tRNA-Ser* (GCU), which lacks a dihydrouridine (DHU) stem. The secondary structure of two ribosomal RNA genes (rRNAs) is predicted based on previous insect models. Twelve types of tandem repeats and two stem-loop structures are detected in the control region, and two stem-loop structures may be involved in the initiation of replication and transcription. Additionally, phylogenetic analyses based on mitogenomes suggest that *Harpalus* is an independent lineage in Carabidae, and is closely related to four genera (*Abax*, *Amara*, *Stomis*, and *Pterostichus*). In general, this study provides meaningful genetic information for *Harpalus sinicus* and new insights into the phylogenetic relationships within the Carabidae.

## 1. Introduction

Mitochondria exist in eukaryotic cells and are inherited maternally, they are associated with energy metabolism, disease, aging, and apoptosis [1]. Insect mitogenomes are characterized by several features shared with most eukaryote mitogenomes, such as a double-strand circular structure, the small genome size (approximately 14–21 K), conserved gene content (13 PCGs, 22 tRNAs and 2 rRNAs), and a lack of extensive recombination [2,3,4]. The complete mitogenome can provide genomic features such as complete genome size, the relative position of genes, RNA secondary structure, and replication and transcription of control patterns [5,6]. Its characterization can help us explore evolutionary patterns across species. Additionally, complete mitogenomes are commonly used to provide a deep-level phylogenetic resolution [7,8].

*H. sinicus* belongs to the Insecta, Coleoptera, Carabidae, and *Harpalus* taxonomic classes, which are widely distributed in mainland China. The *Harpalus* species are omnivorous, preying on the seed of wheat and millet, as well as some small invertebrates such as planthopper and aphids [9]. Adult *Harpalus* species overwinter in soil, stone, and deposits. Overwintering adults begin to have activities in early April of the following year. They mate to spawn during mid-April to mid-May. Males successively die after mating, while females do not die until early July [10]. Morphologically, the body of *H. sinicus* is black and bright with a length of 10–12 mm. They have a tan beard, antennae, and feet, and the lateral edge of the pronotum is brown, and the segmental venter of the body is either black, brown or red in some parts [9]. One feature they share with other Carabidae species is that their foreleg tibiae have a groove bearing a comb of hair for cleaning their antennas. Similar to other Carabidae species, their common habitats are under the soil, on the surface, in the sand of ponds and rivers, in the trees and bushes, or among rocks [11]. The Carabidae is a big family of beetles, containing over 40,000 species around the world, with approximately 1750 species in China [11,12]. However, up to now, only 15 complete mitogenomes from the Carabidae have been reported in GenBank, and no mitogenome of *Harpalus* has been reported before this study. The available data of phylogenetic analyses based on complete mitogenome is significantly limited in the Carabidae.

To fill the gap of genetic information on *Harpalus* and to provide deep-level phylogenetic resolution within Carabidae, we report the complete mitogenome of *Harpalus sinicus*. We analyze the gene content and nucleotide composition of the mitogenome. We find an OL-like region in the mitogenome. We calculate the codon usage and evolutionary dynamics of all PCGs. The secondary structure of all RNA genes is predicted and analyzed. Two stem-loop structures are detected in the control region. Furthermore, deep-level phylogenetic analyses are performed based on complete mitogenomes of the Carabidae species. These results will increase the understanding of *Harpalus* and contribute to further phylogenetic analyses in the Carabidae.

## 2. Materials and Methods

### 2.1. DNA Sequencing and Genome Assembly

An adult *H. sinicus* is collected from Nanchang city, Jiangxi Province (115.85° E, 28.68° N), China. The voucher specimens are deposited in Tianjin State Key Laboratory of Modern Chinese Medicine, Tianjin University of Traditional Chinese Medicine, under the voucher number of H201908. Specimens are preserved in 100% ethanol after being collected and then kept in a refrigerator at −20 °C. Total DNA is extracted using fresh leg muscle tissue from *H. sinicus*. The processes of total DNA extraction and mitochondrial DNA amplification are based on our previous study [13].

### 2.2. Genome Sequencing, Assembly, and Annotation

The DNA library is constructed using fragments with an average insert size of 350 bp. Then DNA is sequenced using the Ion Torrent Personal Genome Machine (Research and Development Center of Traditional Chinese Medicine, Tianjin, China) with a sequencing error rate of 1.5%. Raw sequencing reads generated in FASTQ format are assessed with the FastQC software package [14], and low-quality reads (<Q30) are excluded from further analyses. Then the high-quality sequencing reads are assembled with “de novo assembly” model in IDBA software [15], the generated contigs and scaffolds with K-mer length of 40 are mapped to the mitogenome of a Carabidae species (GQ344500) using a CLC Genome Assembler (v11.0.1, CLC Inc, Aarhus, Denmark) with default parameters. Finally, the generated circular consensus is confirmed via mapping to raw data. The mitogenomic structure is checked by making a comparison with the result from the MITObim software [16]. The complete mitogenome of *H. sinicus* has been deposited into GenBank with accession number MN310888. The annotation of mitogenome is initially performed using the MITOS web server [17] and is confirmed by comparison with annotations from other Carabidae species (Table S1). Additionally, the tRNAs are confirmed using the tRNAscan-SE Web Server [18]. The circular mitogenome map is depicted using OGDRAW [19].

### 2.3. Sequence Analysis

Codon usage and base composition are calculated by MEGA version X [20]. The nonsynonymous (Ka) and synonymous (Ks) substitution rates are deduced from the data of PCGs sequences alignment of two species (*H. sinicus* and *Damaster mirabilissimus mirabilissim*). The program yn00 from the package PAML [21] is used to calculate the nonsynonymous (Ka) and synonymous (Ks) substitution rates of PCGs. The secondary structure of tRNAs is inferred by the MITOS web server [17] with the genetic code of 05-invertebrate. Secondary structure predictions for 16S rRNA (*rrnL*) and 12S rRNA (*rrnS*) are based on the previously reported insect models of *Suwallia teleckojensis* [22] and *Panaorus albomaculatus* [23]. The OL-like structure and two stem-loop structures are inferred using the Mfold web server [24]. The Tandem Repeats Finder server [25] is used to detect tandem repeats with default settings.

### 2.4. Phylogenetic Analysis

In this study, mitogenomes of 15 Carabidae species and two outgroup species as shown in Table S1 are used to evaluate the phylogenetic relationships within the Carabidae. The shared 13 PCGs are extracted, aligned separately, and recombined to construct a matrix using PhyloSuite_v1.1.15 [26]. Maximum likelihood (ML) phylogenies are inferred using IQ-TREE [27] under the model automatically selected by IQ-TREE (‘Auto’ option in IQ-TREE) for 5000 ultrafast [28] bootstraps, as well as a Shimodaira–Hasegawa-like approximate likelihood-ratio test [29]. Bayesian Inference (BI) phylogenies are inferred using MrBayes 3.2.6 [30] under JC + I + G model (2 parallel runs, 2,000,000 generations), in which the initial 25% of sampled data are discarded as burn-in. The final trees are visualized in the Interactive Tree Of Life [31].

## 3. Results and Discussion

### 3.1. The Organization and Nucleotide Composition of the Mitogenome of H. sinicus

**Figure 1:**
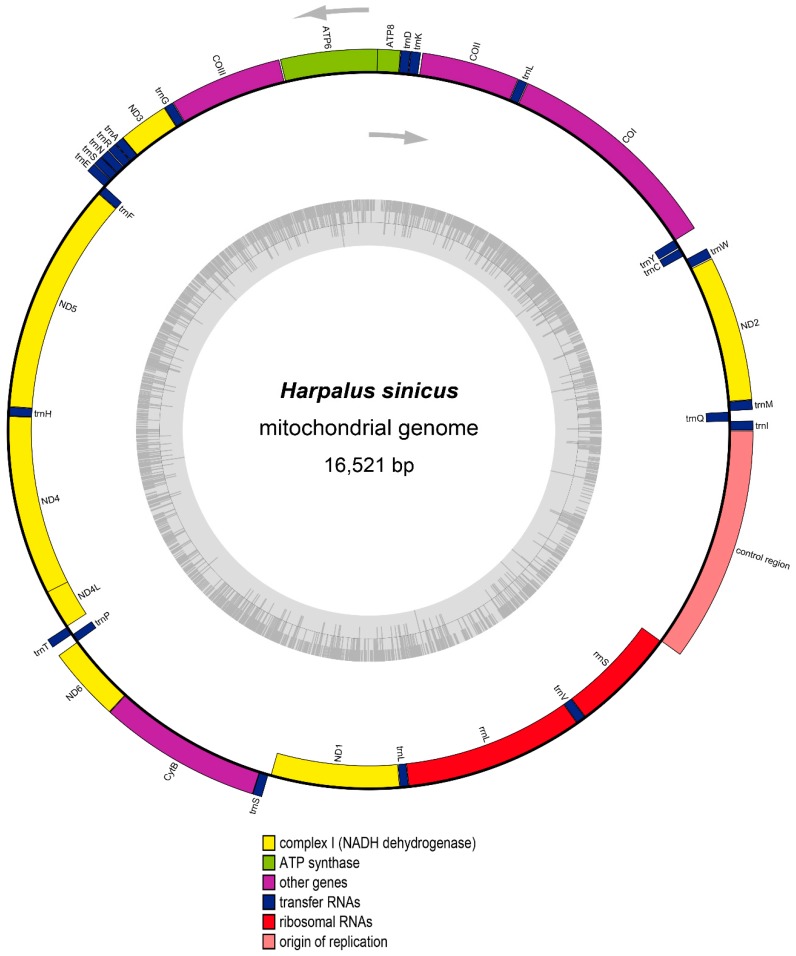
Gene map of the complete mitogenome of *H. sinicus*. Genes outside the circle are located on the J-strand, whereas those inside the circle are located on the N-strand.

The complete mitogenome of *H. sinicus* is 16,521 bp in length, exhibiting a typical double-strand circular structure as shown in Figure 1. Based on mapping contigs on the template of mitogenome GQ344500, the mitogenome contained 37 genes (13 PCGs, 22 tRNAs, 2 rRNAs) with 22 genes located on the majority strand (J-strand) and the remaining 15 genes encoded on the minority strand (N-strand) (Table 1). The mitogenome is relatively compact, with genes overlapping at eight gene junctions. The mitogenome has a total of 91 and 34 nucleotides in spacers and overlaps, respectively, with the longest spacer of 28 bp occurring between *tRNA-Ser* and *ND1*, and the longest overlaps of 7 bp occurring between *ATP8/ATP6* and *ND4L/ND4* (Table 1). The nucleotide composition of the *H. sinicus* is as follows: A = 41.2%, T = 39.4%, C = 10.9%, and G = 8.5%, with the A + T content of 80.6%. The highest A + T content is observed in the control region (88.6%), showing a significant base composition bias toward A and T. Overall, the genome structure and nucleotide composition exhibit typical features of insect mitogenomes [6,32,33].

**Figure 2:**
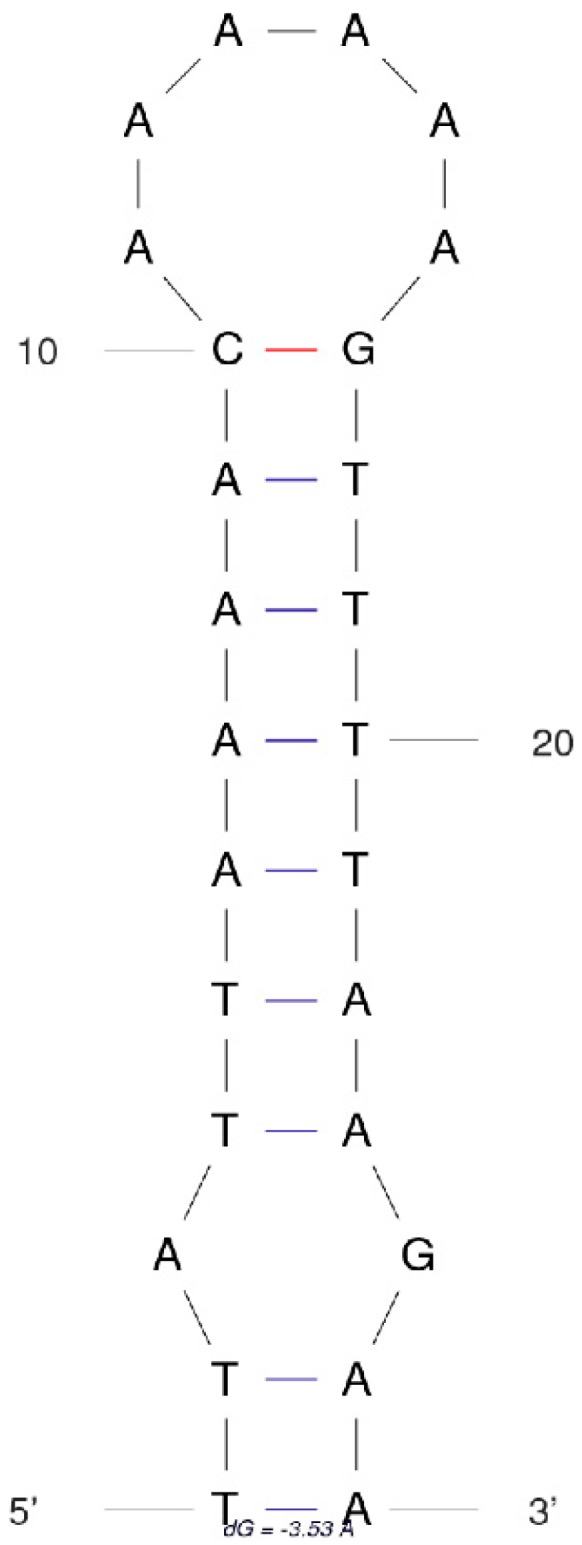
Stem-loop hairpin structure of origins of light-strand replication (OL)-like region in *H. sinicus* mitogenome.

We find an interesting phenomenon that the long insert between *tRNA-Trp* and *tRNA-Cys* can be, from the location and the size, similar to the mitochondrial origins of light-strand replication (OL) that exists in vertebrate mitogenomes [34,35,36,37,38]. The light-strand replication fork forms at OL and proceeds to synthesize the complementary light-strand DNA in vertebrate mitogenomes [39]. We check the sequences and find an OL-like region that can form a stem-loop hairpin structure (Figure 2). The stem-loop hairpin structure of OL-like region is 26 bp in length (location: 1324–1349), including a stem of nine pairs of complementary bases and one pair of mismatched bases. The above features are similar to vertebrate OLs, such as a stem-loop hairpin structure, a length of approximately 30 nucleotides, and a stem of about 10 pairs of complementary bases. Furthermore, we find that the loop consists entirely of A’s (Figure 2). A previous study indicates that PolyA stretches are important for binding mitochondrial DNA polymerases to activate the OL in human mitogenome [40]. The similarity of sequence of this loop and that of the human mitogenome OL loop region implies that it may have the same function for binding mitochondrial DNA polymerases, as indicated in another study [41]. Our study firstly reports the OL-like region in insect mitogenomes, and we suggest that more studies are required to check if it is widely distributed and if it has certain functions in insect mitogenomes.

### 3.2. Protein-Coding Genes

In the mitogenome of *H. sinicus*, all PCGs start with standard ANT start codons except the *COI* gene, which initiates with TTG as the start codon (Table 1). The phenomenon is previously observed in other insect mitogenomes [23,42]. There are 12 PCGs that end with a complete termination codon and 9 PCGs that end with TAA. Three genes (*ND3*, *CytB*, and *ND1*) are terminated with TAG. The *ND5* gene is terminated with an incomplete stop codon A. The incomplete termination codon is commonly used in insect mitogenome due to the presence of post-transcriptional polyadenylation at the 3′ end of mRNA [43].

**Figure 3:**
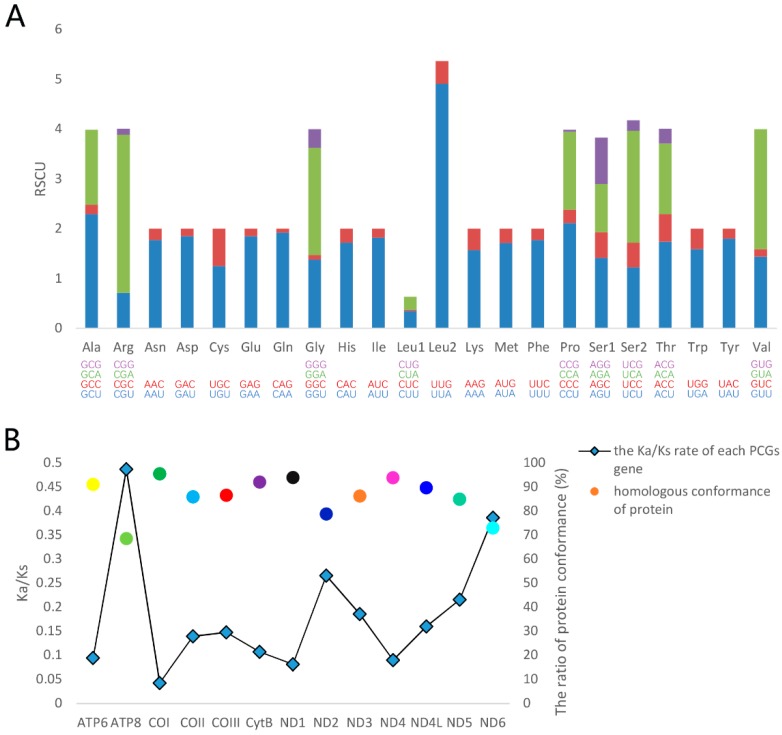
Analyses of protein-coding genes (PCGs) in *H.sinicus*. (**A**) Relative synonymous codon usage (RSCU) of 13 PCGs. (**B**) Protein conformance and evolutionary dynamics of 13 PCGs, *Damaster mirabilissimus mirabilissim* (GQ344500) is used as the reference.

The relative synonymous codon usage (RSCU) reflects the phenomenon of codon usage bias in the genome [44]. Among the codons corresponding to each amino acid, A + T-rich codons have the highest codon usage rate in most amino acids, indicating a strong bias toward A + T-rich codons (Figure 3A). Additionally, the three most frequently used codons are UUA (4.91) for Leu2, CGA (3.18) for Arg, and GUA (2.41) for Val. We calculate the homologous conformance and the ratio of Ka/Ks for each of the PCGs (Figure 3B). Both *H. sinicus* and *Damaster mirabilissimus mirabilissim* belong to the Carabidae, and they share the ancestral genes in mitogenomes. So, Ka/Ks are evaluated using *Damaster mirabilissimus mirabilissim* as a reference. The *COI* gene has the highest conformance value of 95.9%, whereas the *ATP8* gene exhibits the lowest conformance value of 68.5%. Most genes have values that exceeded 80%. The results indicate the evolutionary conservation of PCGs in mitogenome, which can also be observed in the calculations of the ratio of Ka/Ks. The Ka/Ks ratios for all PCGs are less than 1, indicating that these genes are evolving primarily under purifying selection [45]. The lowest Ka/Ks ratio for *COI* gene indicates strong purifying selection and evolutionary conservation in cytochrome c oxidase [46], which results in the *COI* gene often being used as a DNA marker [47]. On the contrary, the *ATP8* gene has the highest Ka/Ks ratios, showing the highest evolutionary among PCGs, which implies that the *ATP8* gene can be used to evaluate intraspecific relationships [48].

### 3.3. Transfer RNAs

**Figure 4:**
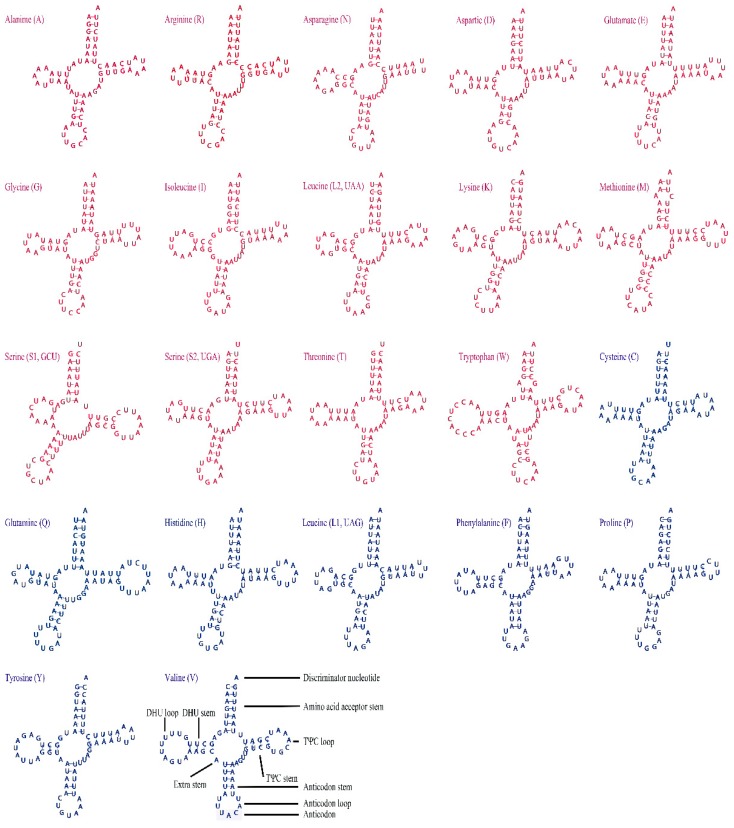
Secondary structure of 22 tRNAs in the *H. sinicus*. The tRNAs located on the J-strand are red, while those located on the N-strand are dark blue.

There are 22 tRNAs in the mitogenome of *H. sinicus*, 14 tRNAs are located on the J-strand and the remaining 8 tRNAs are encoded on the N-strand (Figure 4 and Table 1). All tRNAs can be folded into the typical cloverleaf secondary structures except the *tRNA-Ser* (GCU), which lacks a DHU stem, as observed in other insect mitogenomes [23,32]. The length of the tRNAs range from 64 to 73 bp. Most anticodon stems of tRNAs are 5 bp in length, while only one anticodon stem from *tRNA-Asp* is 4 bp in length. Compared with the anticodon stem, the DHU stem (3–4 bp) and T Ψ C stem (4–5 bp) are more variable (Figure 4). A total of 22 mismatched base pairs are identified in 17 tRNAs and all tRNAs on the N-strand have at least one mismatched base pair. Most mismatched base pairs are G−U, which can form a weak bond in tRNAs [49]. RNA editing may be involved in correcting the mismatched base pairs [50].

### 3.4. Ribosomal RNAs

**Figure 5:**
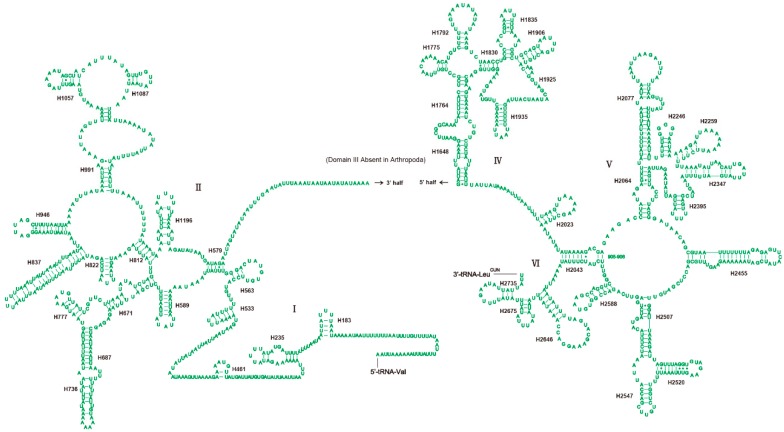
Predicted secondary structure of the mitochondrial *rrnL* in *H. sinicus*. Dashes (−) indicate Watson–Crick base pairing and asterisks (*) indicate non-canonical G−U base pairing.

**Figure 6:**
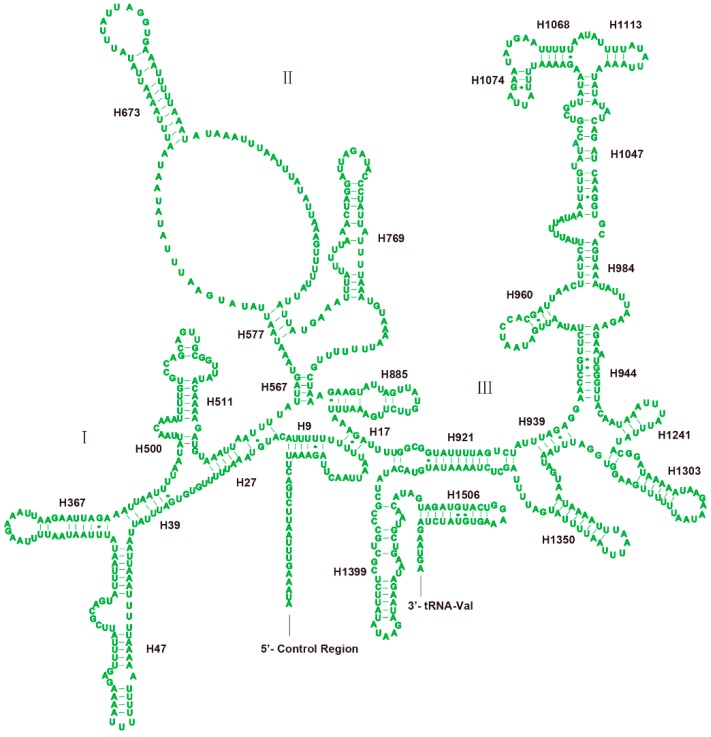
Predicted secondary structure of the mitochondrial *rrnS* in *H. sinicus*. The annotation is the same as for Figure 4.

The *rrnL* and *rrnS* of *H. sinicus* are separated by *tRNA-Val* with no spacer or overlap found as shown in Table 1. The length of *rrnL* and *rrnS* is 1328 and 788 bp, respectively. The secondary structure of *rrnL* is drawn largely based on the insect model of *Suwallia teleckojensis* [22], and the *rrnS* is depicted largely based on the model of *Panaorus albomaculatus* [23]. Both RNAs contain many mismatched base pairs, most of them are G−U. The secondary structure of *rrnL* contains 45 helices and five structural domains (I, II, IV–VI) with domain III absent as in other arthropods [6,33,51] as shown in Figure 5. Compared with *Suwallia teleckojensis*, the structure of stems is more conserved than the loops, and the domains I, II, and IV are more variable than the domain V based on the alignment of sequences and secondary structure of *rrnL*. The secondary structure of *rrnS* contains 27 helices and three domains (Figure 6). Domain I is the most variable region based on the sequence similarity and secondary structure.

### 3.5. Control Region

**Figure 7:**
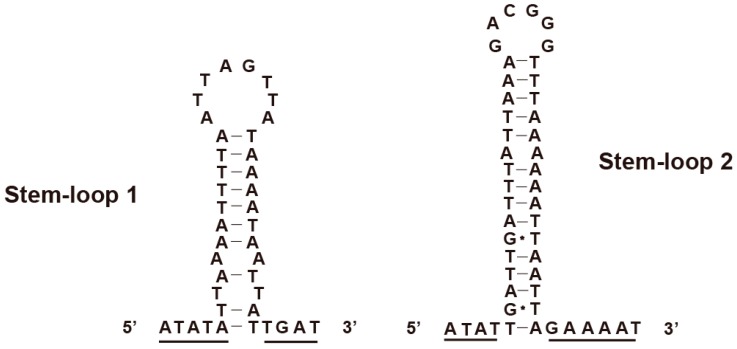
Structure elements found in the mitochondrial control region of *H. sinicus*. The underline indicates conserved sequences.

The control region is previously reported containing elements necessary for the initiation of replication and transcription [52]. The mitochondrial control region of *H. sinicus* is 1651 bp in length (Table 1), with the A + T content up to 88.6%. Due to the high rate of A + T nucleotide composition, the insect control region is also named as an A + T-rich region [2]. The sequences flanking the stem-loop structure are highly conserved in the control region of insect mitogenomes. The 5′ flanking sequences are commonly A+ T-rich (“... TTATA”), while the 3′ flanking sequences appear to have a “G(A)_n_T” motif. Such conserved structures are functionally important to replication origins [53]. Based on the above characterizations, we find two structure elements in the control region as shown in Figure 7. The stem-loop 1 and stem-loop 2 are 40 and 48 bp in length, respectively. Additionally, we detect 12 types of tandem repeats in the control region as shown in Table S2. The length of the repeat units ranges from 14 to 52 bp, and the average copy numbers of incomplete repeats range from 2.4 to 6.8. The corresponding consensus patterns of tandem repeats are shown in Table S2. These tandem repeats can be considered as microsatellite elements that may help to investigate geographic community structure [54].

### 3.6. Phylogenetic Analyses

**Figure 8:**
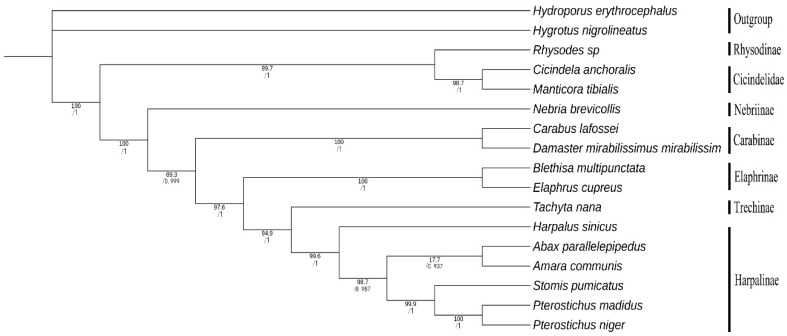
Phylogenetic trees of 15 Carabidae species based on 13 mitochondrial PCGs. Numbers refer to ML bootstrap proportions (above) and Bayesian posterior probabilities (below).

We conduct phylogenetic analyses based on 13 shared PCGs from 15 Carabidae species, and two Dytiscidae species are used as the outgroup as shown in Table S1. We use complete mitogenomes to evaluate the phylogenetic position of *Harpalus* within the Carabidae for the first time as shown in Figure 8. Most nodes in the ML and BI trees have high support values (bootstrap proportions ≥ 89.3, posterior probabilities ≥ 0.937), whereas nodes in the clade *Abax* + *Amara* have significantly low support values (17.7) in the ML analyses. BI and ML analyses provide identical topologies that *Harpalus* is an independent lineage and is closely related to four genera (*Abax*, *Amara*, *Stomis*, and *Pterostichus*) from the subfamily, Harpalinae. Five genera of Harpalinae are clustered as (*Harpalus* + ((*Blethisa* + *Elaphrus*) + (*Stomis* + *Pterostichus*))). In the Harpalinae, the following clades are consistent with a previous study [55]: *Stomis* + *Pterostichus* and *Abax* + *Amara*. Additionally, we recover the clade *Blethisa* + *Elaphrus* and confirm the placement of Rhysodinae as sister to Cicindelidae, as indicated by analysis of previous studies [56]. In our analyses, the clade of each subfamily within the Carabidae is strongly supported, but the position of each subfamily is incongruent with analyses from previous studies [55,56]. Unstable phylogenetic relationships may result from inadequate mitogenome data. We suggest that more mitogenomes are required to resolve the phylogenetic relationships within the Carabidae.

## Figures and Tables

**Table 1:** Organization of *H. sinicus* mitogenome.

Gene	Strand	Location	Size (bp)	Start Condon	Stop Codon	Anticodon	Spacer (+)/Overlap (−)
tRNA-Ile	J	1–68	68			GAT	0
tRNA-Gln	N	66–137	72			TTG	−3
tRNA-Met	J	143–211	69			CAT	5
ND2	J	212–1231	1020	ATA	TAA		0
tRNA-Trp	J	1237–1309	73			TCA	5
tRNA-Cys	N	1338–1402	65			GCA	28
tRNA-Tyr	N	1409–1476	68			GTA	6
COI	J	1469–3010	1542	ATT	TAA		−8
tRNA-Leu	J	3013–3077	65			TAA	2
COII	J	3079–3762	684	ATG	TAA		1
tRNA-Lys	J	3773–3843	71			CTT	10
tRNA-Asp	J	3844–3911	68			GTT	0
ATP8	J	3912–4073	162	ATC	TAA		0
ATP6	J	4067–4744	678	ATG	TAA		−7
COIII	J	4751–5539	789	ATG	TAA		6
tRNA-Gly	J	5541–5606	66			TCC	1
ND3	J	5607–5960	354	ATT	TAG		0
tRNA-Ala	J	5959–6024	66			TGC	−2
tRNA-Arg	J	6025–6090	66			TCG	0
tRNA-Asn	J	6093–6157	65			GTT	2
tRNA-Ser	J	6158–6224	67			GCT	0
tRNA-Glu	J	6226–6292	67			TCC	1
tRNA-Phe	N	6291–6357	97			TGG	−2
ND5	N	6359–8086	1728	ATT	A-		1
tRNA-His	N	8087–8155	69			GTG	0
ND4	N	8155–9495	1341	ATG	TAA		−1
ND4L	N	9489–9785	297	ATT	TAA		−7
tRNA-Thr	J	9788–9853	66			TGT	2
tRNA-Pro	N	9856–9919	64			TGG	2
ND6	J	9921–10,445	525	ATT	TAA		1
CytB	J	10,446–11,585	1140	ATG	TAG		0
tRNA-Ser	J	11,584–11,651	68			TGA	−2
ND1	N	11,669–12,619	951	TTG	TAG		17
tRNA-Leu	N	12,621–12,684	64			TAG	1
rrnL	N	12,685–14,010	1326				0
tRNA-Val	N	14,013–14,085	73			TAC	2
rrnS	N	14,086–14,873	788				0
Control region		14,874–16,524	1651				0

## Supplementary Materials

Supplementary materials can be found at https://www.mdpi.com/2073-4425/10/9/724/s1. Table S1: NCBI accession number. Table S2: tandem repeats.
